# Are There Any Cardioprotective Effects or Safety Concerns of Erythropoietin in Patients With Myocardial Infarction? A Systematic Review

**DOI:** 10.7759/cureus.25671

**Published:** 2022-06-05

**Authors:** Wilford Jean-Baptiste, Amina Yusuf Ali, Bithaiah Inyang, Feeba Sam Koshy, Kitty George, Prakar Poudel, Roopa Chalasani, Mastiyage R Goonathilake, Sara Waqar, Sheeba George, Lubna Mohammed

**Affiliations:** 1 Research, California Institute of Behavioral Neurosciences & Psychology, Fairfield, USA; 2 Neurology, California Institute of Behavioral Neurosciences & Psychology, Fairfield, USA; 3 Internal Medicine, Chitwan Medical College of Medical Science, Chitwan, NPL; 4 Research, California Institute of Behavioral Neurosciences & Psychology, fairfield, USA; 5 Pediatrics, California Institute of Behavioral Neurosciences & Psychology, Fairfield, USA; 6 Internal Medicine, California Institute of Behavioral Neurosciences & Psychology, Fairfield, USA

**Keywords:** coronary heart disease, erythropoietin effects, erythropoietin, stemi, myocardial infarction

## Abstract

Myocardial infarction (MI) is a global cause of morbidity and mortality. MI is the outcome of a chronic process termed atherosclerosis, a buildup of fatty and other substances called plaques inside the coronary vessels, causing hardening and thickening of the arterial wall. Erythropoietin (EPO) is a pleiotropic cytokine released mainly by the kidneys in adults. Besides its well-known erythropoietic functions, EPO possesses anti-apoptotic, mitogenic, and angiogenic effects. This review aims to analyze the strength of any therapeutic or protective value of EPO on the heart and safety concerns regarding its administration in MI individuals.

This systematic review was performed based on Preferred Reporting Items for Systematic Reviews and Meta-Analyses (PRISMA) 2020 guidelines. Four databases (PubMed, PubMed Central, Google Scholar, and Sciences Direct) were employed to search for articles published in the last 10 years. Focused studies were relevant articles in the English language, trials, reviews, meta-analyses, and studies with a control group. Following the quality assessment process, nine studies were eligible and hence were included in the review consisting of six randomized controlled trials and three systematic reviews and meta-analyses. Contrary to preclinical studies, EPO administration did not significantly have notable effects on mortality, major adverse cardiovascular events, or infarction size reduction. Significant left ventricle ejection fraction amelioration was not appreciated either. However, EPO seems to reduce the incidence of post-MI arrhythmias.

## Introduction and background

The most prevalent type of heart disease is coronary heart disease (CHD) [1]. In the United States, it is estimated that 8.4 million adults aged over 20 years have a myocardial infarction (MI), while someone has a heart attack every 40 seconds [2,3]. Hypoxic-ischemic heart or MI, one of the most common cardiovascular diseases, is a leading cause of morbidity and mortality [4]. Until 2030, it is believed that CHD will remain the most common cause of death worldwide [4].

MI is an acute process, exhaustively explored, and is preceded usually by atherosclerosis, which is a phenomenon that restricts blood flow, thus reducing the delivery of oxygen to tissues and subsequently causing tissue necrosis [5]. Primarily, myocardial necrosis is attributed to the degree of cardiac ischemia and secondarily by the ischemia-reperfusion injury and ultimately contributing to the size of infarction and left ventricle remodeling [6,7]. Since the ischemia-reperfusion injury is not easily preventable, ST-elevation myocardial infarction (STEMI) prognosis remains poor even though there has been immense advancement in clinical treatment such as percutaneous coronary intervention (PCI) [6].

Erythropoietin (EPO) is a hormone produced by the kidneys and the liver in adults and during fetal development, respectively, while secreted in response to hypoxic conditions where its primary function is to regulate the plasma hemoglobin level and oxygenation of tissues [8,9]. For instance, it is achieved by fostering the production of mature red blood cells via acting at EPO receptors (EPORs) on the erythroid progenitor cells [4]. Beyond the well-known hematopoietic effects of EPO, this hormone also acts in non-hematopoietic tissue, operating essentially as a pleiotropic cytokine [10,11]. In fact, in the cardiovascular system, there is evidence that EPORs are present at the surface of cardiomyocytes and endothelial cells [12]. In animal studies, administration of EPO intravenously has numerous effects including cell protection against ischemia, anti-apoptotic, vascular endothelial cell proliferation, and neovascularization promotion [9,10]. Partially ascribed to EPO's anti-apoptotic and angiogenic properties, a decrease in MI size, myocardial fibrosis, and amelioration in left ventricle function was observed [7,10].

In preclinical studies, the cardioprotective effects of EPO after MI have been investigated and confirmed [13]. Nonetheless, the efficacy of EPO in reflecting the same cardioprotective effects in humans has been inconsistent and controversial in several randomized controlled trials (RCTs) [13]. Some studies were able to find some cardioprotective impacts, whereas various did not. Therefore, this systematic review is aimed to analyze the evidence of any cardioprotective effect and safety concerns of EPO after MI focusing on humans.

Methods

This systematic review was performed based on the Preferred Reporting Items for Systematic Reviews and Meta-Analyses (PRISMA) 2020 guidelines [14].

Information Sources and Search Strategy

For this review, in April 2022, a meticulous search was conducted in the following databases: PubMed, PubMed Central, Google Scholar, and Sciences Direct. The key terms employed in the search engines were Myocardial infarction, STEMI, and Erythropoietin/Erythropoietin effects, and the Medical Subject Heading (Mesh) strategy was used mainly in PubMed. A composite of the databases and search strategies can be found in Table 1.

**Table 1:** Search strategy for different databases and their search result

Databases	Keywords	Search strategy	Filters	Search result
PubMed	Myocardial infarction, heart attack, cardiac infarction, coronary thrombosis, heart infarct, acute coronary, erythropoietin, EPO, epoetin	(Myocardial Infarction OR Heart attack OR cardiac infarction OR Coronary thrombosis OR heart infarct OR acute coronary OR ( "Myocardial Infarction/blood"[Majr] OR "Myocardial Infarction/chemically induced"[Majr] OR "Myocardial Infarction/chemistry"[Majr] OR "Myocardial Infarction/metabolism"[Majr] ) AND ((y_10[Filter]) AND (ffrft[Filter]) AND (humans[Filter]))) AND (erythropoietin OR EPO OR epoetin OR ( "Erythropoietin/administration and dosage"[Majr] OR "Erythropoietin/adverse effects"[Majr] OR "Erythropoietin/agonists"[Majr] OR "Erythropoietin/analogs and derivatives"[Majr] OR "Erythropoietin/blood"[Majr] OR "Erythropoietin/chemistry"[Majr] OR "Erythropoietin/deficiency"[Majr] OR "Erythropoietin/metabolism"[Majr] OR "Erythropoietin/organization and administration"[Majr] OR "Erythropoietin/physiology"[Majr] OR "Erythropoietin/supply and distribution"[Majr] OR "Erythropoietin/therapeutic use"[Majr] OR "Erythropoietin/toxicity"[Majr] ) AND ((y_10[Filter]) AND (ffrft[Filter]) AND (humans[Filter]))) - 77	Free full text, 10 years cutoff, humans	77
PubMed Central	Effects of erythropoietin, myocardial infarction	Effects of erythropoietin AND myocardial infarction – 6,784	Open access, 10 years cutoff	3735
Google Scholar	Effects of erythropoietin, myocardial infarction, cardiac attack, STEMI	“Effects of erythropoietin” AND “myocardial infarction” OR "cardiac attack" OR "STEMI" – 2,130	10 years cutoff	1040
Sciences Direct	Effects of erythropoietin, myocardial infarction	Effects of erythropoietin AND myocardial infarction – 5,565	Review articles, research articles, 10 years cutoff, medicine and dentistry, pharmacology, toxicology, and pharmaceutical, open access and open archives	303

Inclusion and Exclusion Criteria

Inclusion criteria comprised trials, reviews, meta-analyses, studies with a control group, and papers published in the last 10 years. Additionally, articles related to medicine and dentistry; biochemistry; genetics and molecular biology and pharmacology; and toxicology and pharmaceutical science were taken into consideration. After a careful perusal of titles and abstracts, only free full-text studies in the English language that might meet the inclusion criteria mentioned herein were retrieved. Exclusion criteria included papers published over 10 years, thesis, case reports, pediatric studies, editorials, papers unrelated to EPO and cardiovascular systems, and animal studies.

Data Collection and Analysis

Two researchers independently extracted the relevant data according to the inclusion and exclusion criteria. Full articles were analyzed extensively, and any discrepancies were reevaluated and reassessed by both researchers and discussed to reach common ground. The final studies were categorized into RCTs and systematic reviews and meta-analyses. Items garnered to describe RCTs eligible studies include first author and year, country, sample size in both EPO and control groups, male percentage, mean years of age, EPO doses, comparator arm, and follow-up period. Systematic review and meta-analyses were described similarly to RCTs except for the country and comparator arm.

Final Findings Assessment

The outcome emphasized the primary and secondary endpoints for each final included study. Additionally, any cardioprotective effect in the EPO and the control group regarding changes in left ventricle ejection fraction (LVEF), left ventricle end-systolic and end-diastolic (LVESV and LVESV), incidence of arrhythmias, cardiac remodeling, major adverse cardiovascular events (MACE), and EPO safety were all assessed.

Result

Study Selection and Quality Assessment

The search among the databases concluded with 5,155 publications. A total of nine articles were deleted automatically from Google Scholar. The articles' citations were imported to Endnote where they were sorted alphabetically. Later, automatically and manually, a total of 251 duplicates were removed. Finally, based on their titles, abstracts, and relevancy to the research topic, 24 free full-text articles were sought for quality appraisal and thus eligibility.

**Figure 1:**
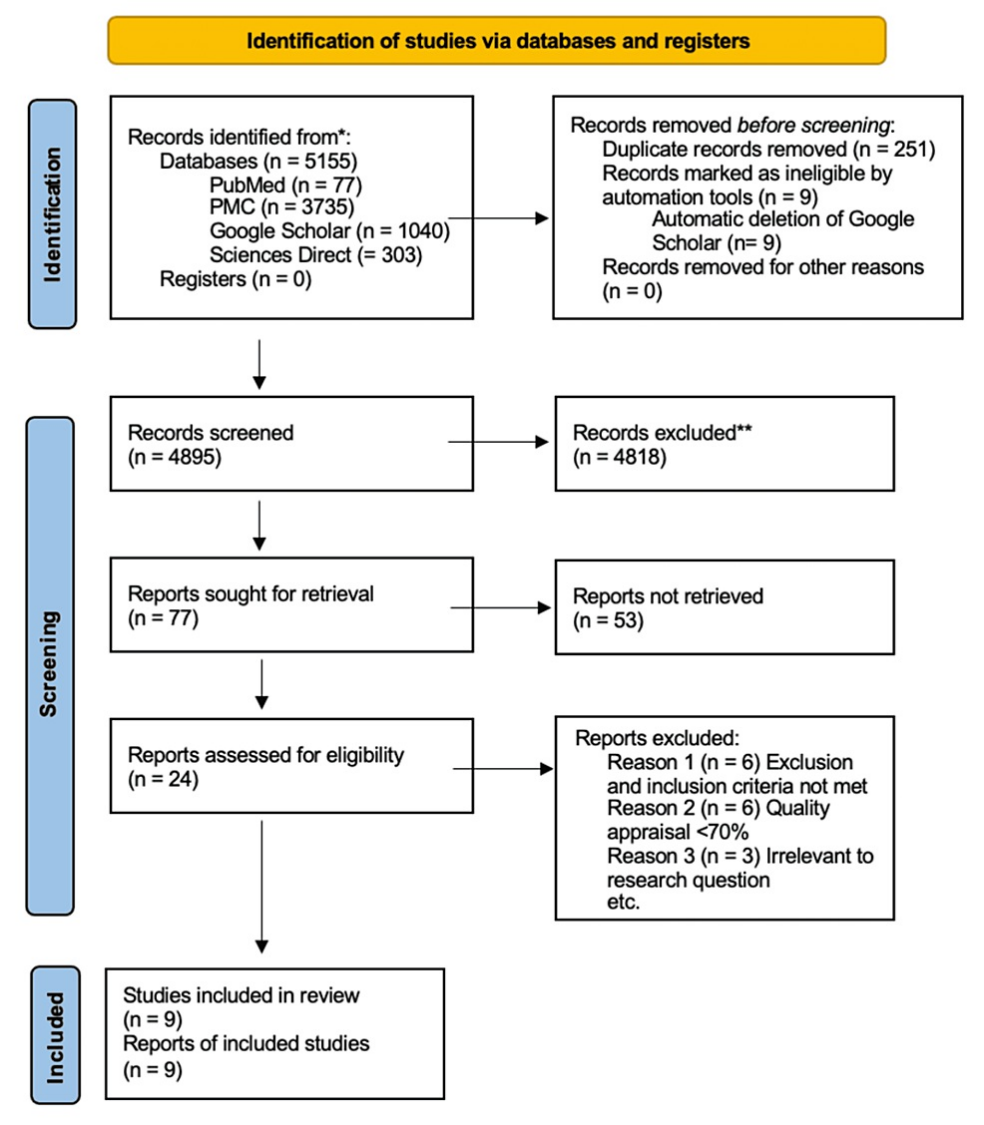
PRISMA 2020 flowchart of the databases and studies PRISMA, Preferred Reporting Items for Systematic Reviews and Meta-Analyses [14]

The quality assessment tools drawn up during the process are the following: Cochrane risk-of-bias tool (Rob 2) for RCTs, Assessment of Multiple Systematic Reviews (AMSTAR 2) for systematic reviews and meta-analyses, and quality appraisal tool for cross-sectional studies (AXIS) [15-17]. The quality assessment was conducted by two individuals independently. For articles to be eligible, they must score at least 70% or get an overall low risk in risk-of-bias if appropriate, depending on the quality assessment tool. Following the quality appraisal, nine studies yielded from the process resulting in six RCTs and three systematic reviews and meta-analyses. A summary of the study selection using the PRISMA flow diagram and the quality assessment can be found in Figure 1 and Table 2, respectively.

**Table 2:** Quality appraisal tools of final studies LR, low risk; RCTs, randomized controlled trials; RoB, risk of bias

Quality appraisal tool	Type of study	Number of studies	No of included studies and RoB/score
Cochrane risk-of-bias tool (Rob 2) [15]	RCTs	9	6: LR
Assessment of Multiple Systematic Reviews (AMSTAR 2) [16]	Systematic reviews and meta-analyses	4	3: ≥70%
Appraisal tool for cross-sectional studies (AXIS) [17]	Cross-sectional Studies	2	0: ≥70%

Basal Characteristics of Eligible Studies

The nine studies included 2,837 patients in the experimental group (EPO group) and 2,749 in the control group (placebo or standard medical treatment). The overall mean age ranged from 48 to 70 years. The composite studies had EPO doses ranging from 3,000 to 100,000 IU and were administered intravenously with one exception, which was intracoronary. The follow-up period ranged from 24 hours to 5 years. Different types of EPOs were used, and the time of administration after PCI varied between the studies. The comparator arm mainly drew up standard medical treatment, saline, or placebo. The basal characteristics of the included studies of RCTs, systematic reviews, and meta-analyses are detailed in chronological order in Table 3 and Table 4, respectively.

**Table 3:** Basal characteristics of included RCTs of STEMI/acute myocardial infarction patients EPO, erythropoietin; RCTs, randomized controlled trials, rhEPO, recombinant human erythropoietin; STEMI, ST-elevation myocardial infarction

First author (year)	Country	EPO group	Control group	Mean age, years	Male %	Dose of EPO and time of administration	Comparator arm	Follow-up
Fokkema et al. (2013) [18]	The Netherlands	263	266	60.3	78.1	IV 60,000 IU of epoetin alpha; 3 hours after PCI	Standard medical treatment	1 year
Gholamzadeh et al. (2015) [8]	Iran	20	20	52.8	72.5	33,000 IU of rhEPO; immediately after PCI	Saline	24 hours
Steppich et al. (2017) [6]	Germany	68	70	60.6	78	3 doses of IV 33,000 IU of rhEPO beta; immediately, at 24 hours, and 48 hours after PCI	Placebo	5 years
Minamino et al. (2018) [10]	Japan	68 and 70	60	60.9	85.8	IV 12,000 or 6,000 IU of epoetin beta; within 6 hours of PCI	Placebo	6 months
Orii et al. (2018) [7]	Japan	32	29	69.5	80	IV 12,000 of epoetin beta; immediately after PCI	Saline	8 months
Seo et al. (2019) [13]	Republic of Korea	40	40	59.5	83.75	Intracoronary 300 μg of darbepoetin alpha; simultaneously with PCI	Saline	4 months

**Table 4:** Basal characteristics of systematic reviews and meta-analysis AMI, acute myocardial infarction; EPO, erythropoietin; RCTs, randomized controlled trials, STEMI, ST-elevation myocardial infarction

First author (year)	Study design	Study population	No and type of included study	EPO group	Control group	Mean age years range	EPO-dosing range	Follow up range
Li et al. (2012) [19]	Systematic review and meta-analysis	STEMI	9 RCTs	607	619	48 to 62	3,000 to 100,000 IU	1 to 6 months
Wen et al. (2013) [20]	Systematic review and meta-analysis	STEMI	7 RCTs	612	620	-	30,000 to 100,000 IU	1 to 6 months
Ali-Hassan-Sayegh (2015) [12]	Systematic review and meta-analysis	STEMI/AMI	15 RCTs	1057	1025	48 to 63	3,000 to 100,000 IU	1 month to 1.5 years

Outcome

The outcome of the nine studies was divided into "findings" and "EPO safety" because the latter has been a focus of every study. All of the studies mentioned their primary and secondary endpoints except for two studies that only stated their primary endpoint. The outcome is detailed in Table 5.

**Table 5:** EPO cardioprotective effects and safety of included studies in MI patients ACVB, aortocoronary venous bypass; HCT, hematocrit; HF, heart failure; Hgb, hemoglobin; mHR, heart rate; EPO, erythropoietin; LV, left ventricle; LVEDV, left ventricle end-diastolic volume; LVEF, left ventricle ejection fraction; LVESV, left ventricle end-systolic volume; MACE, major adverse cardiovascular events; MI, myocardial infarction; PCI, percutaneous coronary intervention; RCTs, randomized controlled trials

First author (year)	Study design	Primary endpoint	Secondary endpoint	Findings	EPO safety
Li et al. (2012) [19]	Systematic review and meta-analysis	Change in LVEF, MRI assessment of cardiac function	LVESV, LVEDV	Slight improvement in LVEF (1.38%). No significant effects on LVESV, LVEDV, and infarct size.	Relatively safe
Fokkema et al. (2013) [18]	RCTs	Vital status, reinfarction, target vessel revascularization, stroke	All-cause mortality, HF incidence	No significant effects on the composite endpoint	Safe
Wen et al. (2013) [20]	Systematic review and meta-analysis	Mean of LVEF, infarct size by MRI	Cardiac death, MACE	Limited cardioprotective effects of EPO	Safe
Ali-Hassan-Sayegh (2015) [12]	Systematic review and meta-analysis	Incidence on HF, re-MI, stroke, thrombosis, MACE, mortality, LVEF, LVESV, LVEDV, infarct size, creatine kinase serum level		No significant effect on the composite endpoint, except a decrease in HF incidence in high-dose EPO (>30,000 IU)	Safe
Gholamzadeh et al. (2015) [8]	RCTs	Incidence of arrhythmia in 24 hours after PCI	Hematologic and hemodynamic evaluation after 2 weeks	High dose of EPO (>30,000 IU) has significantly reduced the incidence of arrhythmia. No significant effects of EPO on HCT, Hgb, systolic and diastolic blood pressure, and HR	Safe
Steppich et al. (2017) [6]	RCTs	Incidence of MACE, death, recurrent MI, stroke, ACVB, target revascularization		EPO has no impact on the composite endpoint and thus does not improve long-term clinical outcome	Caution concerning EPO with MI
Minamino et al. (2018) [10]	RCTs	LVEF improvement	Safety and efficacy of EPO	No amelioration of EPO on LVEF. No significant impact on MACE and non-cardiac death.	Safe
Orii et al. (2018) [7]	RCTs	Effects of EPO on myocardial viability, effects of EPO on coronary circulation, effects of EPO on cardiac remodeling	Safety analysis	EPO had a beneficial effect on coronary microvascular dysfunction and a beneficial effect on left atrium remodeling	Safe
Seo et al. (2019) [13]	RCTs	Myocardial infarct size	Infarct size, proportion of salvaged myocardium, post-infarct remodeling, cardiac death, non-fatal MI, stent thrombosis, HF	Intracoronary EPO did not mitigate the myocardial infarct size. No impact on post-ischemic LV remodeling	Safe

## Review

Discussion

The overall effects of EPO on the cardiovascular system and MI have been compelling in experimental studies; however, multiple clinical studies have been controversial. This section of the systematic review explores the mechanism of EPO at a molecular level in the fundamental cells of the cardiovascular system to gauge its beneficial effects. Moreover, this section examines the detrimental effects of EPO administration in MI individuals to scope its general safety in such patients. Ultimately, the therapeutic value of EPO in clinical studies in the study population in terms of changes in mortality, arrhythmias’ incidence, thromboembolic events, MACE, LVEF, inflammatory biomarkers, and infarction size have also been discussed.

*Molecular Mechanism of EPO and the Cardiovascular System*

**Figure 2:**
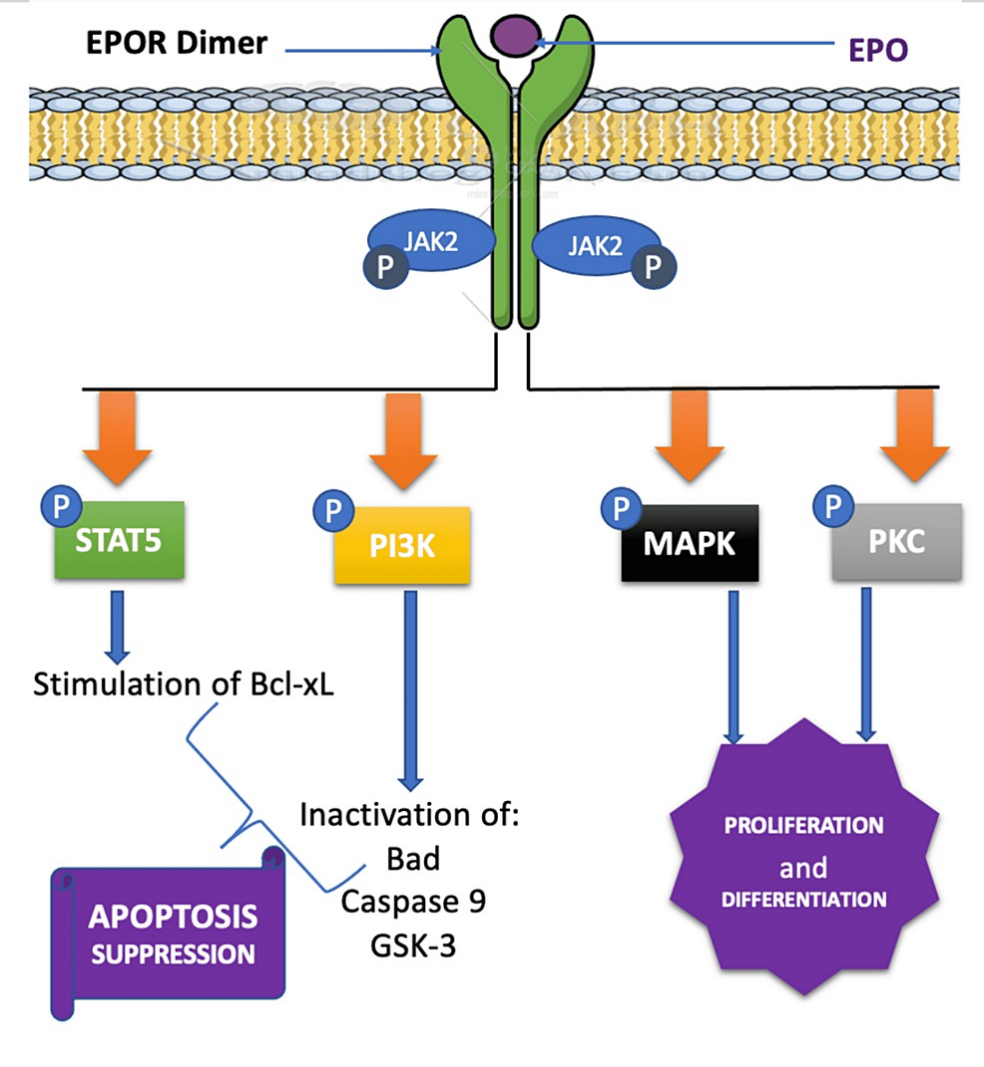
Signaling pathways targeted by EPO-EPOR binding Figure created by the author, Jean-Baptiste, using Mind the Graph. Bad, BCL2-associated agonist of cell death; Bcl-xL, B-cell lymphoma-extra large; GSK3, glycogen synthase kinase 3; JAK 2, Janus kinase 2; MAPK, mitogen-activated protein kinase; PI3K, phosphoinositide 3-kinases; PKC, protein kinase C; STAT5, signal transducer and activator of transcription

EPOR is distributed among multiple systems and expressed on the surface of plethoric cell types including, but not limited to, cardiomyocytes, vascular, endothelial smooth muscle cells, hematopoietic cells, myoblast, macrophages, lymphocytes, enterocytes, and proximal tubule epithelium [4]. EPOR is a transmembrane receptor (type 1 cytokine); when the EPO and EPOR interaction occurs, three signaling pathways are involved [4]. The classical pathway prompts involvement and phosphorylation of Janus kinase (JAK2) and activation of stat5, a transcription factor, and followed ultimately by upregulation of Bcl-xL, an anti-apoptotic gene; the second pathway is predominated by JAK2 kinase-dependent NF-kB, which activates this kinases complex and eventually cause expression of anti-apoptotic genes; the last pathway requires activation of other survival kinases such as mitogen-activated protein kinases (MAPK) and phosphatidyl-Inositol 3 kinases and Akt signaling pathway to bolster the rates of proliferation, survival, and differentiation of stimulated cells [4]. EPO-EPOR binding strongly enhances the proliferation and migration of human umbilical vein endothelial cells; however, smooth muscles did not observe such results and interestingly pointed out the difference among the cell types [9]. Indeed, the anti-apoptotic, proliferation, differential, and survival properties of EPO can have several benefits on different tissues promoting cell regeneration, anti-inflammatory, and proangiogenic features, especially during ischemic injuries. EPO signaling pathways are illustrated in Figure 2.

One endothelial cell has around 30,000 EPOR molecules on its surface, although with a lower affinity for EPO compared to erythroid progenitor cells where they have lower EPOR molecules but a higher affinity for EPO [21]. For instance, the administration of recombinant human EPO (rhEPO) in healthy and advanced kidney failure patients has been shown to stimulate the proliferation and differentiation of endothelial progenitor cells (EPCs) [21]. These authors, in vitro, demonstrated that the efficacity of EPO to mobilize the EPCs was dose-dependent through activation of the Akt signaling pathway [21]. However, in patients with heart failure who were on chronic rhEPO, changes in the number of EPCs were not evident, and the cohesive and proliferation features of EPCs were augmented [21]. On the other hand, rhEPO has been observed to foster the production of two molecules, endothelin 1 (ET-1) and plasminogen activator inhibitor 1 (PAI1), both leading to an increase in vascular resistance resulting in hypertension seen in patients with chronic kidney disease (CKD) [21]. EPCs play a pivotal role in neo-angiogenesis and endothelial repair. Therefore, these effects of EPO on regulating EPCs explain its cytoprotective effects on the cardiovascular system. Nevertheless, most studies could not confirm the mitogenic effects of EPO on human vascular smooth muscles since they are the main cells in the medial layer and are paramount in sustaining the integrity of the arteries.

**Figure 3:**
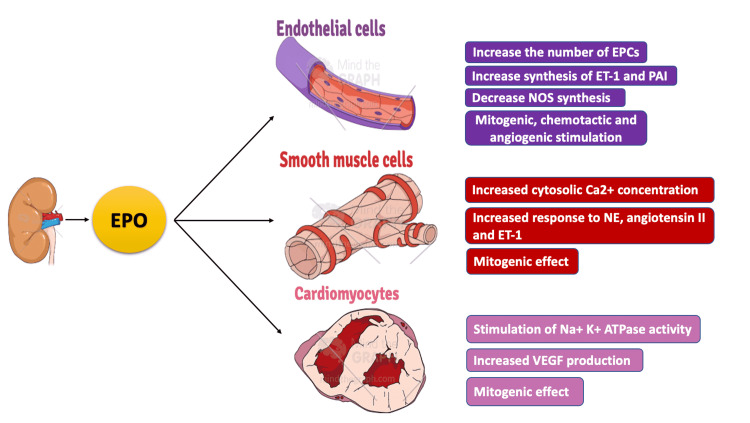
EPO’s effects on the cardiovascular system Figure created by the author, Jean-Baptiste, using Mind the Graph EPCs, endothelial progenitor cells; ET-1, endothelin 1; NE, norepinephrine; NOS, nitric oxide synthase; PAI, plasminogen activator inhibitor 1; VEGF, vascular endothelial growth factor

EPO plays an essential part in cardiac embryonic development in experimental studies on mice [22]. EPO and EPOR interaction on the surface of cardiomyocytes can preclude the apoptosis of cardiomyocytes [23]. Indeed, deletion of the EPO or EPOR gene during embryonic days contributes to severe ventricular hypoplasia in part due to impaired erythropoiesis and the loss of EPO-proliferating effect on cardiac myocytes, anti-apoptotic, and EPCs mobilization features, which result in vascular malformation [22]. A summary of EPO’s effects on the cardiovascular system can be found in Figure 3.

Adverse Effects of EPO Administration and MI

Serious concerns regarding the adverse effects of EPO-stimulating agents (ESAs) have been raised, mainly in CKD patients and treated cancer patients [24]. The commonality in these studies on ESAs safety is EPO’s ability to stimulate prothrombotic cytokines such as prostaglandin F2 alpha and thromboxane [25]. In contrast, EPO seems to decrease the release of prostacyclin in cultured endothelial cells with an increase in the number of circulating platelets and their reactivity [25,26]. In acute coronary syndrome, the platelet number is decreased, and no increase in platelet activity in patients receiving EPO analogs was observed [26]. On the other hand, in patients in non-acute coronary settings, thromboembolic events were more likely to be observed [26].

The common adverse effects of EPO include seizures, increased hemoglobin and hematocrit, hypertension, thromboembolic events, and cancerogenic effects [8]. However, multiple studies have not found notable adverse effects in patients receiving EPO in patients with MI [8,12,18,20,27].

People may be reluctant to administer EPO analogs in patients with AMI due to the dread of the aforementioned deleterious effects, particularly in MI thrombogenic nature. However, the included studies, except for one study, showed that a moderate EPO dose has no adverse effects in MI patients. Thus, the safety of EPO may be dose-dependent and more research is warranted regarding that.

Effects of EPO on MI

The formation of coronary collateral vessels remarkably reduces mortality because these new vessels deliver oxygenated blood to make up for coronary arterial occlusion [28]. In patients with chronic coronary artery total occlusion, endogenous serum EPO levels were significantly higher and were associated with better coronary collateral vessel development independent of hemoglobin concentration level [29]. However, one cross-sectional study on the association between serum EPO level and the formation of coronary collateral vessels found anemia to be a potent stimulator of coronary collateral vessel formation instead of serum EPO level in coronary artery disease [25]. More importantly, in terms of all-cause mortality, reinfarction, and thromboembolic events, FokkemaMarieke et al.’s study including STEMI individuals, one year after PCI, concluded that epoetin alfa had no long-term effects [18]. Similarly, the REVIVAL-3 study by Steppich et al. demonstrated with a five-year RCT (the longest follow-up to date) an insignificant long-term clinical effect in STEMI patients treated with epoetin beta [7].

Inflammatory and cardiac disease biomarkers YKL-40, IL-6, pro-BNP, and CK-MB were evaluated in patients undergoing coronary artery bypass graft surgery [11]. Administration of EPO before the procedure reduced the biomarkers' serum levels compared with the control group, confirming the hypothesis of EPO's anti-inflammatory properties [11]. Contrastingly, Foroughi et al. also stated in a study published by Mocini et al. that the authors could not reveal EPO efficacy in reducing troponin 1 and CK-MB serum levels to protect against ischemic reperfusion injury [11]. G-CSF, a stem cell mobilizer, has been shown to regulate CD34/KDR cells, which are peripheral blood stem cells; furthermore, a combination of Darbepoetin with G-CSF exhibited a higher rate of mobilization of proangiogenic cells in patients with AMI [30]. Different routes of EPO administration in MI, including epicardial, intraperitoneal, and intramyocardial delivery, have been evaluated [31]. In both rat and human mesenchymal stem cells, epicardial EPO seems to be the most effective at targeting the important intracardiac regenerative molecular mechanisms and pathways in the early post-ischemic heart [31].

Ventricular tachycardia (VT) and ventricular fibrillation contribute considerably to morbidity and mortality in patients with STEMI undergoing PCI. RhEPO has been shown to significantly lower the occurrence of arrhythmias in this population by more than two-fold when comparing the EPO group to the placebo group [8]. On the contrary, Minamino et al. observed no impact of EPO on the incidence of VT, atrial fibrillation/flutter, and sick sinus syndrome [10].

Orii et al. noted in Japanese patients with anterior MI that in the EPO group of patients, the transmural infarction extension was lower in the acute phase, with a significantly higher coronary flow velocity reserve at eight months [7]. This study reflects the beneficial effect of EPO on coronary microvascular circulation [7]. Moreover, the EPO group had significantly higher LVEF, and the left atrium volume was lower at eight months [7]. In a meta-analysis of multiple RCTs, there was an amelioration of 1.38% in LVEF, with no significant effects on LVESV, LVEDV, or infarction size [24]. Compared with that, before PCI, a high dose of intracoronary darbepoetin alfa (>90,000 IU of epoetin alfa) was administered, and no notable effect on infarct size and post-ischemic LV remodeling was observed [13]. A similar finding in a meta-analysis by Wen et al. concluded limited heart protection after STEMI on a high dose of EPO (>30,000 IU) [20].

The timing of EPO administration was immediately after induced coronary thrombosis in animal studies; EPO doses ranged from 2,500 to 5,000 IU per kg in rats versus 430-860 IU per kg in humans [6]. These might, for instance, explain the discrepancy in outcomes between preclinical and human studies. However, a non-erythrogenic EPO derivative, asialo-rhuEPO, was found to have better cardioprotective effects than rhEPO in shielding the cardiomyocytes against injury and apoptosis [32]. These promising findings regarding asialoerythropoietin may inspire its use as a novel agent for future experimental studies and, subsequently, in humans.

Limitations

The search strategy for the systematic review was limited to four databases where only papers published in the English language in the last 10 years (2012-2022) were included. The study merely analyses free full-text studies and thus may have precluded the inclusion of eligible studies. Moreover, the total number of patients in the EPO and the control group does not reflect unique patients because some RCTs were also present in some systematic reviews. Additionally, the discrepancy in timing of EPO administration after PCI and the dramatic difference in EPO dosing among the studies avert an objective conclusion regarding EPO cardioprotective effects in MI patients.

## Conclusions

EPO administration in MI may reduce the incidence of post-MI arrhythmias, although larger RCTs are needed to confirm such therapeutic effects. In contrast, EPO administration in MI did not significantly have beneficial effects in reducing mortality, MACE, infarction size, or amelioration in LVEF, as reported herein, differing from animal studies. This contrast between preclinical and human studies may be due to dosage differences, the timing of EPO administration, or intricate differences between human and animal tissues. Thus, recommendations include the use of preclinical dosage of asialoerythropoietin, a non-erythrogenic EPO derivative, which will circumvent the deleterious effects of the rhEPO or synthetic EPO at a supraphysiologic level. In addition, administration of EPO at the time of the first MI symptoms will reflect the experimental studies more accurately. Finally, with the EPO dose range reported in the included articles, an overwhelming majority of the studies consider EPO to be safe in MI.
